# Valgus stability is enhanced by flexor digitorum superficialis muscle contraction of the index and middle fingers

**DOI:** 10.1186/s13018-020-01640-7

**Published:** 2020-03-30

**Authors:** Shota Hoshika, Akimoto Nimura, Norimasa Takahashi, Hiroyuki Sugaya, Keiichi Akita

**Affiliations:** 1 Shoulder & Elbow Service, Funabashi Orthopaedic Sports Medicine & Joint Center, Funabashi, Chiba Japan; 2 Department of Clinical Anatomy, Graduate School of Medical and Dental Science, Tokyo Medical and Dental University, Tokyo, Japan; 3 Department of Functional Joint Anatomy, Graduate School of Medical and Dental Sciences, Tokyo Medical and Dental University, Tokyo, Japan

**Keywords:** Ulnar collateral ligament, Ultrasound, Flexor digitorum superficialis, Overhead sports

## Abstract

**Background:**

Flexor digitorum superficialis (FDS) muscle provides dynamic stabilization and medial elbow support for ulnar collateral ligament (UCL). The FDS contraction significantly affects the medial joint distance (MJD) through grip contraction. However, it remains unclear whether FDS activity alone contributes to medial elbow stability, or together with the activation of the flexor digitorum profundus during grip contraction, and which finger’s FDS is the main contributor to elbow stability. We investigated the resistive effects of isolated FDS contraction in individual fingers against valgus stress in the elbow joint using stress ultrasonography (US).

**Methods:**

We investigated 17 healthy males (mean age, 27 ± 5 years). Valgus stress US was performed using the Telos device, with the elbow at 30° flexion. MJD was measured for each arm during 3 separate conditions: at rest (unloaded), under valgus load (50 N) (loaded), and under valgus load with FDS contracted in individual fingers (loaded-contracted).

**Results:**

MJD was significantly longer when loaded (5.4 ± 0.4 mm) than unloaded (4.1 ± 0.2 mm, *P* = 0.007) or loaded-contracted (4.6 ± 0.3 mm, *P* = 0.003) for each finger. When loaded-contracted, MJD differed statistically between the index and ring fingers (*P* = 0.03) and between the middle and ring fingers (*P* = 0.04). However, the difference between the index and middle fingers was not statistically significant (*P* = 0.08).

**Conclusions:**

Individual FDS contraction, particularly of the index and middle fingers, contributes most to stabilization against valgus stress. Thus, injury care programs should incorporate FDS exercises of these fingers.

## Background

The prevalence of ulnar collateral ligament (UCL) injury and resultant operations in young baseball pitchers and professionals is increasing disturbingly [1, 2]. In order to avoid reconstruction surgery and its associated year-long recovery, improvement of preventive care programs is necessary [2]. The anterior bundle of the UCL is the primary restraint to valgus load at the medial elbow [3]. During the throwing motion, the tensile load on the UCL has been estimated to exceed its failure strength, leading to injury [4]. The flexor-pronator muscles (FPMs) are secondary dynamic stabilizers and have been considered to exert a protective effect against UCL injury [5–13]. However, the preventive arm care programs did not assess the effect of the finger flexors [14, 15].

Anatomical studies indicate that the FPMs lie in a good position to protect the UCL [6, 8]. Biomechanical studies report that the flexor digitorum superficialis (FDS) muscle plays the greatest role among the FPMs as an active stabilizer against valgus stress [10, 11, 13, 16]. Ultrasonographic (US) studies report that the FDS has significant effects on medial joint distance (MJD) through grip contraction [14, 17]. However, there have been concerns relating to the assessment of FDS activity. First, it remains unclear whether FDS activity alone contributes to medial elbow stability, or together with the activation of the flexor digitorum profundus (FDP) during grip contraction [18]. Second, it remains unclear which finger’s FDS contributes most to elbow stability.

This study aimed to evaluate MJD at rest, under valgus load, and under valgus load with isolated FDS contraction of each finger using stress US. We hypothesized that FDS activity alone contributes to medial elbow stability, and that FDS contraction in one specific finger provides dynamic stability against valgus stress.

## Methods

### Design

This study was a repeated measures analysis to assess changes in MJD during separate elbow loading conditions. The dependent variable was MJD, and the independent variables were the loading conditions and FDS contraction of each finger. The participants’ dominant and nondominant elbows were subjected to rest (unloaded), valgus load (loaded), and valgus load with individual FDS contraction (loaded-contracted). These conditions were tested by separate examinations of the index, middle, and ring fingers.

### Participants

Seventeen physically active individuals aged 22–38 years (mean age, 27 ± 5 years) were recruited within our institute from December 2017 to November 2018. Only male participants were enrolled, to mimic the state of an adult male baseball player. Participants with any of the following were excluded: (1) current pain or injury in the upper extremities, (2) previous UCL tear or elbow dislocation, (3) previous surgery in the upper extremities, and (4) previous participation in overhead sports. Institutional review board approval was obtained and written informed consent was obtained from all participants prior to enrollment.

### Imaging technique

**Fig. 1:**
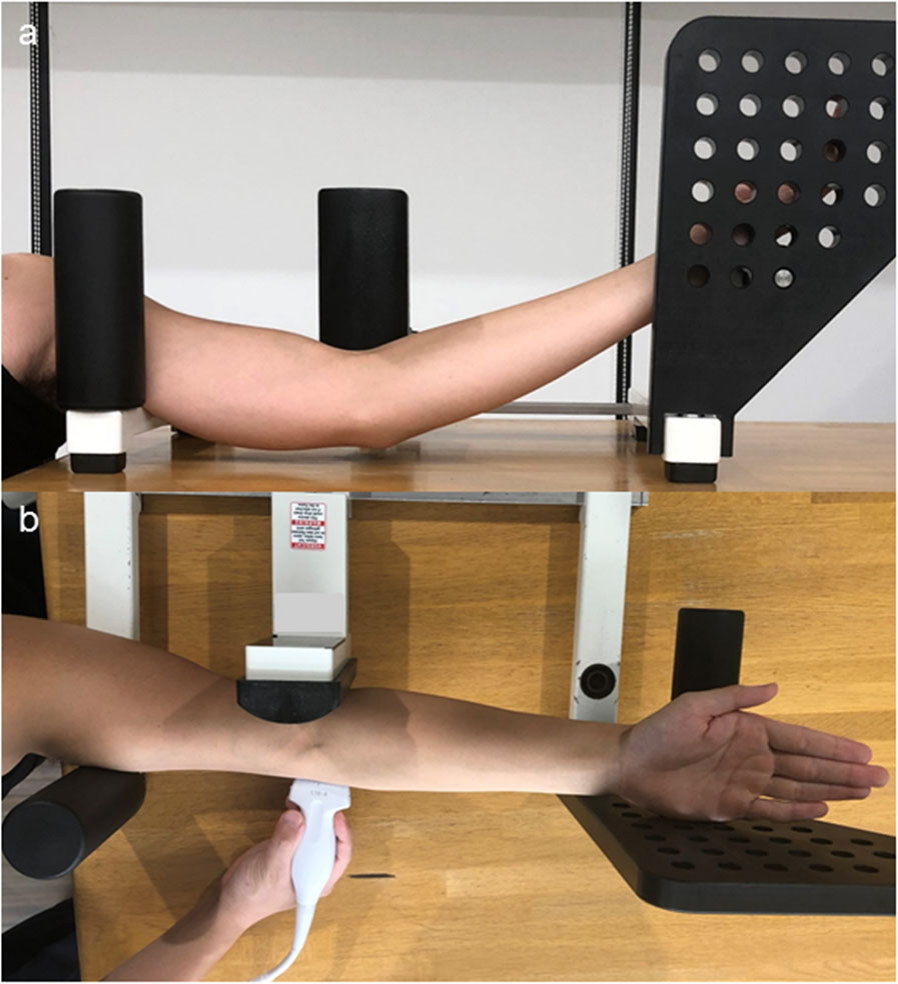
The positioning of the elbow on the Telos stress device. **a** Subject sitting on a chair with the shoulder at 60° abduction, the elbow at 30° flexion, and the forearm in a supination position. **b** The ultrasonographic images are obtained from the medial side of the elbow. 50 N of valgus stress is applied to the lateral side of the elbow by the Telos device

All US scans were performed by an experienced orthopedic surgeon (SH) using a SNIBLE (KONICA MINOLTA, Chiyoda, Japan) scanner with an 11-MHz linear transducer. Each participant was placed in a sitting position on a chair with the shoulder at 60° abduction, the elbow at 30° flexion, and the forearm in supination, similar to that in previous research (Fig. 1a) [19, 20]. All angles were measured using a goniometer, and the transducer was placed on the oblique coronal plane to visualize the MJD; this method is sensitive enough to identify an increase in the MJD when a valgus load is placed on the arm (Fig. 1b) [19–21].

### Stress ultrasonographic examination across the 3 loading conditions

**Fig. 2:**
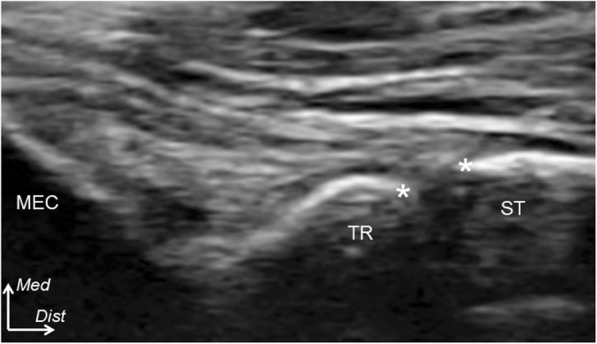
An oblique coronal ultrasonographic examination of the humeroulnar joint. The medial joint distance is measured as the distance between the distal-medial corner of the trochlea (TR) and the proximal edge of the sublime tubercle (ST) (asterisks), in millimeters, from the images in three loading conditions: unloaded, loaded, and loaded-contracted. MEC, medial epicondyle; *Med*, medial; *Dist*, distal

The stress US examination was used to assess the contribution of the FDS contraction of each finger to medial elbow joint stability. The MJD was measured as the distance between the distal-medial corner of the trochlea of the humerus and the proximal edge of the sublime tubercle of the ulna on the oblique coronal image (asterisks in Fig. 2). This was measured on the US screen with the use of electronic calipers with a precision of 0.1 mm. Fixed valgus stress (50 N) was applied using a standardized instrumented device (Telos, Marburg, Germany) to the lateral side of the elbow joint to strain the medial aspect of the elbow (Fig. 1b). This valgus stress of 50 N was selected for two reasons: (1) some participants were unable to tolerate stress of over 50 N and were uncomfortable and (2) this amount of force has been suggested as appropriate for the Japanese population [22]. Using these established methods of collecting images and applying valgus stress, we were able to collect the measurements (mm) of the unloaded, loaded, and loaded-contracted testing conditions [14].

Under the unloaded condition, the participant was placed in the testing position with no valgus stress applied to the elbow joint. The participant was asked to relax completely before an image was taken. Under the loaded condition, the participant was asked to relax while the valgus load was applied, and another image was taken. As a transition took place between the loading conditions, the valgus load was fully removed from the elbow to prevent excessive stress. A 1-min transition period was allowed between the testing conditions.

**Fig. 3:**
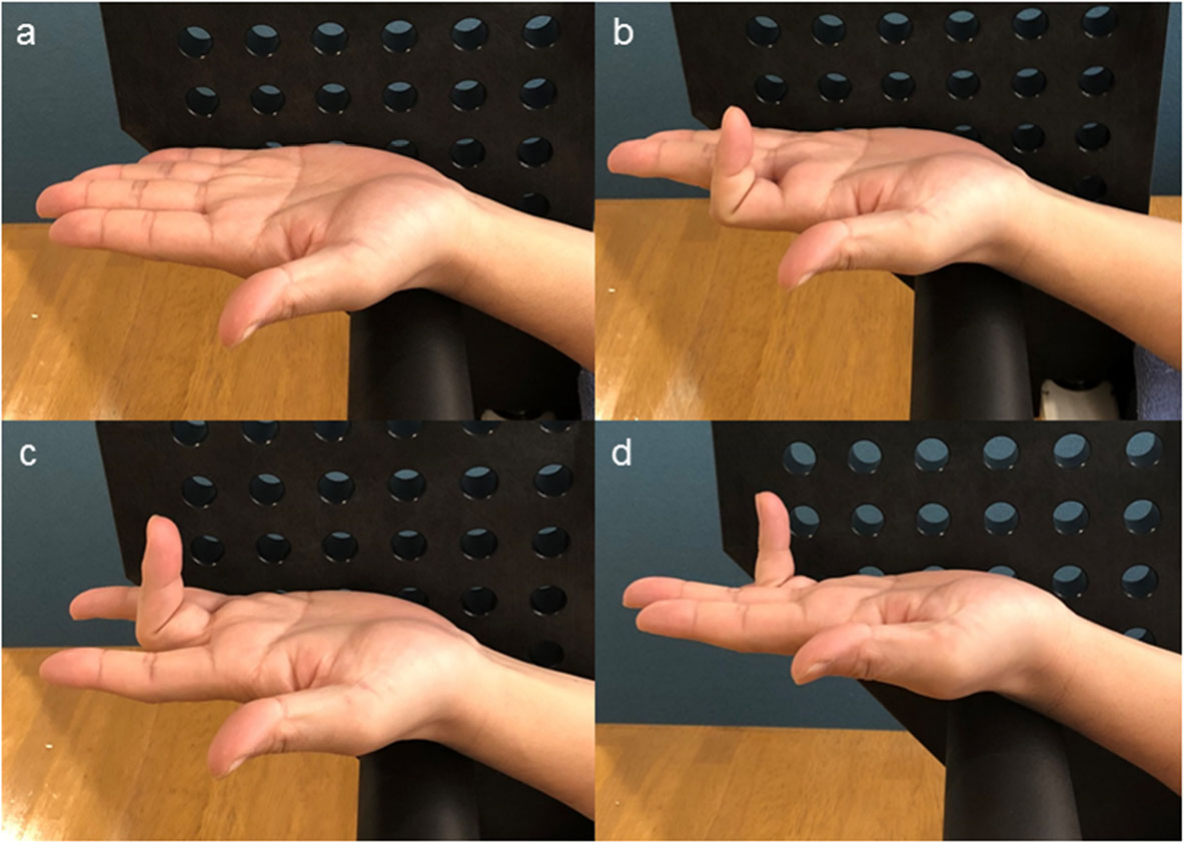
Anterolateral view of each finger for individual flexor digitorum superficialis (FDS) activity. **a** All fingers are kept straight, thus not contracting the FDS. **b** The index finger is flexed at a fixed angle of 90° at the proximal interphalangeal joint and maintained straight and free from any muscle contraction at the distal interphalangeal joint; this is done to only activate the FDS and to eliminate the effect of flexor digitorum profundus contraction of the specific finger. The participants maintain the activation by keeping the finger bent. **c** The FDS activation of the middle finger. **d** The FDS activation of the ring finger

In the loaded-contracted condition, the participant was asked to flex the specified finger and maintain it at a fixed angle of 90° at the proximal interphalangeal (PIP) joint (Fig. 3). To eliminate the effect of FDP contraction, we ensured that the finger was kept free of any tension at the distal interphalangeal (DIP) joint. With the finger in the flexed position, the FDS was activated, leading to its isometric contraction. The final image was taken while the load was applied during muscle contraction. No participants experienced elbow pain during the examination. Data for three trials of the separate loading conditions in each finger were collected and averaged for data analysis. We randomly tested the dominant and non-dominant arms and tested the index, middle, and ring fingers in that order. For interrater reliability, the MJD was evaluated twice at > 4-week intervals. Interrater reliabilities were established and maintained, with interclass correlation coefficients in the acceptable range for all measures (0.90–0.98).

### Statistical analysis

A one-way repeated measures analysis of variance (ANOVA) was performed to assess changes in the MJD for the separate loading conditions in each finger. When the ANOVA indicated statistical significance, a Bonferroni *t* test was performed. In addition, a paired *t* test was applied to compare the MJD between the dominant and non-dominant arms for each finger. A two-sided *P* value of < 0.05 was considered to indicate statistical significance. All analyses were performed with IBM SPSS Statistics 18 software for Windows (IBM Japan, Inc.). A power analysis for the detection of differences between separate loading conditions in each individual finger was conducted using an *α* value of 0.05, an effect size of 0.4 (which was determined according to the results of a preliminary study), and a power of 0.95. The power analysis suggested that 34 elbows were needed to assess the three separate loading conditions for each finger.

## Results

**Fig. 4:**
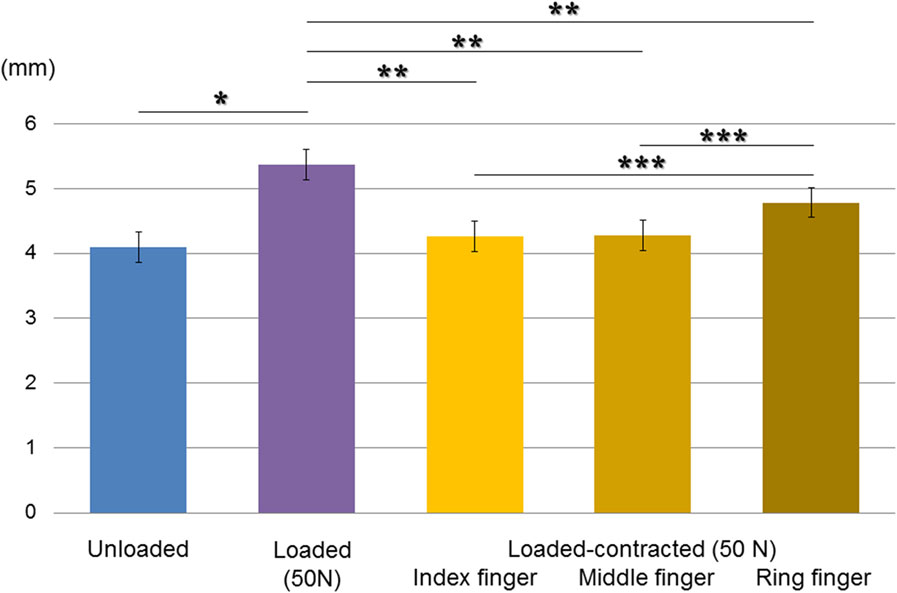
Bar charts showing medial elbow joint distance across 3 loading conditions measured for each finger. Mean values are expressed in millimeters. *Significant differences from loaded (*P* < 0.05). **Significant differences from loaded (*P* < 0.05). ***Significant differences from loaded-contracted of ring finger (*P* < 0.05)

Figure 4 and Table 1 show the values of MJD for each finger and both elbows. MJD was significantly longer when loaded (5.4 ± 0.4 mm) than unloaded (4.1 ± 0.2 mm, *P* = 0.007) or loaded-contracted (4.6 ± 0.3 mm, *P* = 0.003) (Fig. 4). When loaded-contracted, MJD showed no statistically significant difference between the dominant and non-dominant arms for any finger (Table 1). There was a statistical difference in the MJD between the index and ring fingers (*P* = 0.03), and also between the middle and ring fingers (*P* = 0.04), when loaded-contracted (Fig. 4). However, the difference in MJD between the index and middle fingers was not statistically significant (*P* = 0.08) (Fig. 4).

**Table 1:** The bilateral medial elbow joint distance in the loaded-contracted condition for each finger

		Dominant	Non-dominant	*P* value
Finger	Index	4.3 (4.0–4.6)	4.3 (4.0–4.6)	0.59
	Middle	4.3 (4.0–4.6)	4.3 (4.0–4.6)	0.95
	Ring	4.8 (4.6–5.0)	4.8 (4.4–5.2)	0.70

Values are expressed as millimeters (mean (95% CI)). The locations of measurements are demonstrated in Fig. 2

## Discussion

The most important findings of this study were that FDS activity alone contributes to medial elbow stability. In addition, the FDS muscles of the index and middle fingers provide more dynamic stability against valgus stress than that of the ring finger.

Grip strength is an important indicator of hand function, including the functions of the FDS and FDP muscles. A hand dynamometer is used to evaluate the maximum power of the total grip strength of all fingers [23]. However, the FDP is considered a primary finger flexor, generating greater gripping strength than the FDS [24]. A recent biomechanical study demonstrated that the contribution of the FDS and FDP to the grip strength depends on the hand dynamometer angulation and contact area of each finger [18]. In addition, it is difficult to accurately evaluate grip strength in each finger by using the dynamometer. Finally, the grip strength of all fingers could not be associated with the FDS function in each finger. In our study, to obtain isolated FDS activation, the subject was asked to flex the PIP joint while the DIP joint remained quiescent and not flexed in each finger [24]. Clearly, our study showed the contribution of isolated FDS function in each finger to the dynamics of the MJD.

Some anatomical studies reported that the FDS was the individual muscle best suited to provide medial elbow support because of its proximity and relatively large bulk among the FPMs [6, 8]. Current in vitro research recognized that FDS activity had significant effects on MJD using stress US [14, 17]. However, these studies overlooked the importance of assessing FDS activity of individual fingers, as opposed to the activity in all of them collectively, through grip motion; thus, it remained unclear which finger’s FDS contraction contributed most to elbow stability. This study demonstrates that FDS contraction of the index and middle fingers has a larger effect on MJD than that of the ring finger. This explains how FDS activation of the index and middle fingers has a primary role for stabilization of the medial elbow against valgus stress.

The stress US study showed that healthy volunteers displayed 0.7 mm of change in the MJD as measured with non-stress and stress [17]. Another study demonstrated that the change in the MJD under loaded and loaded-contracted conditions of the FDS was 0.2 mm [14]. Based on the previous studies, a minimum of 0.2 mm could be interpreted as a clinically significant difference in the loaded and loaded-contracted conditions compared to the unloaded condition. In the present study, the change in the MJD under loaded and loaded-contracted conditions of the isolated FDS compared to the unloaded condition was 1.3 and 0.8 mm, respectively. Finally, the isolated FDS contraction could have clinically significant effects on the MJD to protect from valgus stress.

Previous studies have reported that the UCL of the dominant elbow in professional baseball pitchers was thicker than that of the non-dominant elbow and its thickness was associated with increased laxity against valgus stress [20, 25]. Recently, Hoshika et al. [26] identified that the anterior bundle of the UCL could be interpreted as part of the tendinous complex, which consisted of the tendinous septum (TS) and the FDS muscle. In other words, UCL thickness could be interpreted as the FDS muscle thickness. Given the anatomical fact, we could hypothesize that UCL thickness will change with medial elbow stabilization by the FDS contraction of each finger. Future research is needed to assess the changes in muscular thickness of the FDS with valgus stability by FDS contraction of each finger using the MJD through the stress US.

Based on these findings, an interpretation of the dynamic stabilizing effect of FDS function in these specific fingers can be described anatomically as follows: At the level of the mid forearm, the FDS muscles of the index and middle fingers are located on the radial side, and the ring and little fingers on the ulnar side. In addition, the FDS muscle of the index finger is located in the superficial layer and that of the middle finger in the deep layer [27]. Hoshika et al. [26] also reported that the tendinous complex might be a pathway of the muscular power of the FPMs to the humeroulnar joint. Taking these anatomical concepts into consideration, the FDS muscles of the index and middle fingers may connect with the TS; thus, these fingers could provide more dynamic stability against valgus stress than that of the ring finger via the TS.

Our study provides clinically relevant information that can be used in the development of a program to prevent UCL injury. Recently, there has been a focus on hand muscle training to prevent throwing injuries as well as to improve performance in generating ball velocity [14, 26, 28]. However, Thrower’s Ten, which has a long history of use by overhead throwing athletes, incorporates multiple strengthening exercises for the shoulder, elbow, and wrist but fails to address the finger flexors [15]. Taking our current results into consideration, the preventive care program could be improved by incorporating finger exercises that focus particularly on the FDS muscles of the index and middle fingers. This study might aid the development of such programs.

The study has the following limitations: First, FDS activity of the little finger was not examined because it has no impact on throwing a ball. Second, we enrolled participants from the general population, and the medial elbow of baseball players may have adapted to chronic medial elbow stress and react differently. Future research in these athletes will allow the assessment of how these characteristics change in a sport-specific population. Third, we did not check any electromyography of the FDS and FDP muscles for the precise assessment of muscle activity in each finger, when cued to perform isolated FDS contraction without the effect of the FDP muscle. Given these limitations, this is the first study to assess MJD during FDS contraction in each finger.

## Conclusions

This study reveals that individual FDS contractions, especially of the index and middle fingers, directly impact elbow dynamics more than that of the ring finger. Finger exercises that focus specifically on these muscles are important for preventing UCL injuries.

## Data Availability

The datasets used and/or analyzed during the current study are available from the corresponding author on reasonable request.

## Abbreviations

ANOVA: Analysis of variance
DIP: Distal interphalangeal
FDP: Flexor digitorum profundus
FDS: Flexor digitorum superficialis
FPMs: Flexor-pronator muscles
MJD: Medial joint distance
mm: millimeter
PIP: Proximal interphalangeal
TS: Tendinous septum
UCL: Ulnar collateral ligament
US: Ultrasonographic
